# Perceived Facilitators and Barriers in Response to a Walking Intervention in Rural Cancer Survivors: A Qualitative Exploration

**DOI:** 10.3390/ijerph15122824

**Published:** 2018-12-11

**Authors:** Lauren J. Frensham, Gaynor Parfitt, Rebecca Stanley, James Dollman

**Affiliations:** 1 School of Psychology Social Work and Social Policy, University of South Australia, Adelaide, SA 5001, Australia; 2 Alliance for Research in Exercise, Nutrition and Activity (ARENA), School of Health Sciences, University of South Australia, Adelaide, SA 5001, Australia; gaynor.parfitt@unisa.edu.au (G.P.); james.dollman@unisa.edu.au (J.D.); 3 Early Start, School of Education, Faculty of Social Sciences, and Illawarra Health and Medical Research Institute, University of Wollongong, Wollongong, NSW 2522, Australia; rstanley@uow.edu.au

**Keywords:** cancer survivorship, walking intervention, motivators, barriers, rural

## Abstract

Physical activity has numerous associated benefits for cancer survivors. Compared to their urban counterparts, rural and remote Australians experience a health disadvantage, including poorer survival rate after the diagnosis of cancer. The purpose of this qualitative study was to (a) investigate factors that motivated or inhibited walking in rural participants during a 12-week intervention and (b) to investigate factors that motivated or inhibited physical activity behavior change three months post-intervention. Ten cancer survivors living in rural areas of South Australia participated in a 12-week computer-delivered walking-based intervention during which they reported daily steps, daily affect, and ratings of perceived exertion. Based on this information, individualized daily step goals were sent to them to increase walking. Following the intervention, participants engaged in face-to-face semi-structured interviews. Interviews were recorded, transcribed and coded using thematic analysis. Participants identified a range of physical, psychological, social, environmental, and organizational motivators and barriers. Participants appreciated the monitoring and support from the research team, but some voiced a need for better transition to post-program and many desired ongoing support to maintain their motivation. Future studies should incorporate strategies to help walking behavior to become more intrinsically motivated and therefore sustained.

## 1. Introduction

As the population of cancer survivors increases, there is a need for interventions that reduce the negative physical and psychological side effects of diagnosis and treatment. Cancer and its treatments are often associated with adverse physical and psychological effects including fatigue, functional impairment, weight gain, sleeping difficulties, and reduction in quality of life [1,2]. These side effects can persist for months or years after treatment, however there is currently little focus on managing these after-effects during survivorship.

Regular physical activity provides a non-pharmacologic strategy for the prevention and/or alleviation of many of these negative effects. Research has consistently shown that physical activity has a positive impact on physiological outcomes (e.g., cardiovascular fitness, physical functioning, immune function, muscle strength, body composition, nausea, and fatigue) and psychological wellbeing (e.g., mood, self-esteem, anxiety, and depression) following a cancer diagnosis [3,4,5]. Furthermore, studies have indicated that physical activity may reduce the risk of cancer recurrence and increase survival rates in breast and colorectal cancer populations [3,4]. Despite these health benefits, and the fact that physical activity has been deemed a safe activity following a cancer diagnosis [5], many cancer survivors do not meet physical activity recommendations [6,7].

People living in rural areas are often perceived to be more active than those living in metropolitan areas due to the physical nature of work such as agriculture, forestry, and fishing [8]. However, according to the Australian Institute of Health and Welfare [9], those living in regional and remote areas were 1.16 times more likely to be sedentary compared with people in metropolitan areas. Surveys in rural Victoria and South Australia showed that only 30% of men and 21% of women were meeting the physical activity guidelines [10]. This may be due to unique features of the rural environment including geographic diversity, social isolation, limited access to facilities, and the burden of travel distances [11]. Cancer survivors living in rural and remote areas are at even higher risk of being physically inactive and experiencing negative effects after treatment than their urban counterparts [12,13]. Different challenges and motivators for engagement in physical activity may exist between metropolitan and rural dwellers; therefore, it is possible that different strategies may be needed in order to overcome these challenges and promote sustained physical activity engagement.

Physical activity interventions have had varying levels of adoption success, however, maintenance over the long term is more challenging to achieve, as the majority of those who start a physical activity program drop out or relapse back to baseline activity levels [14]. While the literature on physical activity maintenance is limited, some evidence suggests that the predictors of adoption are different from those of maintenance. Accordingly, physical activity adoption and maintenance require unique approaches and underlying theoretical frameworks. Identifying specific motivators and barriers to physical activity among rural cancer survivors is important, as it can ultimately lead to effective strategies to facilitate sustained physical activity among this vulnerable population.

The purpose of this qualitative study was to explore rural cancer survivors’ perceptions of the factors influencing their ability to engage in a pedometer-based walking intervention and maintain physical activity behavior change beyond the intervention. Details of the quazi-randomized intervention, Steps Toward Improving Diet and Exercise (STRIDE) protocol, have been published elsewhere [15]. In summary, intervention participants wore a pedometer to monitor their walking and were encouraged to reach daily step goals. These goals were tailored to the individual by taking into consideration physical impairments or restrictions that the participant may have had, as well as how they were feeling each day and their own perceptions of exertion.

The research questions guiding the study were (i) what factors do rural cancer survivors identify as enabling them to engage in and maintain participation in physical activity following the STRIDE intervention? Additionally, (ii) what factors do they see as hindering them to engage in and maintain participation in physical activity following the STRIDE intervention?

## 2. Materials and Methods

### 2.1. Participants

This study received ethical approval from the University of South Australia Human Research Ethics Committee (Application ID: 0000031039). Eleven participants from two rural towns in South Australia who had completed the STRIDE intervention program were contacted via telephone and invited to participate in a face-to-face interview approximately three months after the three-month follow-up period. A total of 10 rural cancer survivors (5 females and 5 males) were interviewed.

### 2.2. Procedure

Participants engaged in the 12-week STRIDE intervention with a 3-month follow-up period [15,16]. Participants were eligible if they were insufficiently active (defined as engaging in less that 20 sessions of exercise over the past month (one session is 30 min duration) determined by The Active Australia Survey [17]; aged 18 years or older; had cancer treated with curative intent; were not currently undergoing treatment; had regular access to the Internet; had sufficient English language skills; satisfied stage one of the Sports Medicine Australia pre-exercise screening system [18]; received approval by their treating doctor to be part of the study; and provided written informed consent. Participants were ineligible if they had metastasis; were pregnant; or had physical or psychological conditions that may have impeded their participation. Participants were randomly assigned to either the intervention or control group. All participants received a pedometer, but only the intervention group had access to the STRIDE website where they reported daily steps, daily affect and ratings of perceived exertion during exercise. Researchers used these variables to individualize daily step goals to increase walking. STRIDE was designed upon Social Cognitive Theory, which posits that physical activity interventions are most effective when they include intrapersonal, social, and environmental mediators [19,20]. The STRIDE website incorporated intrapersonal mediators including goal setting and self-monitoring (a graph provided feedback on progress throughout the intervention). These were aimed to increase self-efficacy. Social mediators included an online forum through which participants shared experiences and provided peer support, and a virtual notice board where community service providers advertised events and activities. Social support has been consistently reported as a predictor of maintained behavior change in lifestyle promotion [21,22]. The website also included information on healthy eating based on the Cancer Council Australia’s nutrition guidelines [23], which in turn are based on recommendations in The Australian Guide to Healthy Eating [24]. Participants could access the website at any time during the 12-week intervention and 3-month follow-up period.

STRIDE also incorporated Self-determination Theory [25], which is considered as a valuable framework to explain motivational processes underlying exercise and sport behavior [26]. The theory suggests that human beings have innate tendencies toward psychological growth and development and strive to master ongoing challenges. Self-determination Theory identifies three innate needs: Autonomy, competence, and relatedness. The STRIDE program encouraged autonomy by allowing participants to choose one of three daily step goals depending on their daily affect. This provided participants with a sense of choice, personal control, and self-endorsement. Competence was encouraged by setting realistic and achievable step goals and by helping identify barriers to physical activity and strategies to overcome them. Relatedness was encouraged by providing a supportive environment for participants (for example, the online forum encouraged participants to share experiences with each other) so participants could experience a sense of connectedness and belongingness with other participants and research personnel.

Concepts from Locke and Latham’s [27] goal setting theory were integrated by encouraging (1) goal acceptance (participants were provided with a workshop on the importance of active lifestyles, thus placing importance on the achievement of their step goals); (2) goal specificity (step goals were tailored to the individual, taking into consideration their affect and physical capability); (3) providing difficult goals (step goals were set high enough to encourage high performance, but low enough to be attainable); and (4) feedback (a graph on the STRIDE website showed weekly step count averages over the 12-week program).

At the conclusion of the program, semi-structured face-to-face interviews were conducted by two trained research staff among rural participants who received the 12-week intervention. The aim of the study was to assess participants’ perceived motivators and barriers to engaging in the program and maintaining regular physical activity beyond the intervention. Theme saturation was reached following 10 interviews. Questions asked during the interview related to the change in walking patterns, perceived motivators for walking, barriers faced, step goals, and physical activity maintenance. Sample questions from the interview included: ‘What contributes to you continuing to be active? Why is it important?’ and ‘What barriers did you face, if any, to your walking?’ Probing questions were used when responses were dichotomous (e.g., yes/no) and more in-depth information was required. Interviews each lasted approximately 45 min. The participants were given a $20 gift voucher in recognition of their time and contribution to the study.

### 2.3. Coding and Analysis

A qualitative descriptive design was used. Interviews were transcribed verbatim. The first author (LF) coded all interviews thematically using NVivo10 qualitative analysis software. All data were analyzed using qualitative content analysis. Most thematic categories were labelled using descriptive terms within the narratives, while some were driven by questions in the interview guide. The first level of coding identified the broad substantive content, for example, motivation to increase physical activity. Subsequent levels of coding involved re-examining the content of these codes to identify commonalities and differences. Categories describing comparable experiences were grouped together under a higher order concept, as recommended by Strauss and Corbin [28]. A second researcher (RS) reviewed and commented on the coding categories. Any discrepancies in coding were resolved between the two coders until agreement was achieved. Finally, the codes were grouped into categories and sub-categories, and reviewed to identify overarching themes.

## 3. Results

### 3.1. Participants’ Characteristics

The socio-demographic and health characteristics of the participants are presented in Table 1.

### 3.2. Qualitative Findings

Three main themes were identified as motivators for physical activity: Physical/psychological benefits, social factors, and website factors. Four main themes were identified as barriers to physical activity: Physical limitations, psychosocial barriers, environmental barriers, and organizational barriers. These themes and their sub-themes are described in Table 2 below.

#### 3.2.1. Perceived Motivators

Factors that motivated participants’ ability to be active were aggregated into physical/psychological, social, and website factors.

##### (1) Physical/Psychological Factors

Participants identified a number of tangible physical benefits that motivated them to continue being physically active. These included improved cardiovascular fitness, increased energy levels, improved body image, and weight loss. Several participants mentioned that they ‘*feel fitter*’ and were more physically mobile due to the increased walking:

*I’m not getting out of breath as much now. I feel better in myself, my skin is quite clear … I feel better in myself*.

(Female, 78, breast cancer)

*I’m fitter, I’d lost weight with the steps and walking up the hills, I plateaued out now a little bit, but I haven’t put on weight*.

(Female, 60, breast cancer)

*I have noticed that by being more active I can actually do more with my knees. So, I can now squat for a little bit of time, and I can now kneel, where I used to never be able to*.

(Female, 62, breast cancer)

Some participants mentioned increased energy levels as a result of the program, which was an important outcome for them. One participant commented:

(I) *don’t seem to get so tired now like I used to before because before I started on this, I’d be really tired by the end of the day and have no motivation and so it’s really helped me that way*.

(Male, 70, prostate cancer)

One participant mentioned that following the STRIDE program, she started boot camp. She commented that her increased walking during the STRIDE program increased her fitness and improved her confidence in ‘tackling’ boot camp:

*… it’s (boot camp) probably something I wouldn’t have tackled before, but now I’m tackling it and crawling on my knees and doing all sorts of things that I don’t think I would’ve done if I hadn’t increased my walking. It helped me, the walking helped me, to start and walk up hills so then I started to feel fitter and things like that. And then I was looking for another challenge so Boot Camp’s a challenge*.

(Female, 60, breast cancer)

##### (2) Social Support

A frequent theme throughout the interviews was that participants enjoyed receiving encouragement and support from the research team during the intervention. The support received was a motivational factor and also kept participants ‘accountable’. The following quote demonstrates externally regulated motivation:

*Somebody’s looking over your shoulder, making sure you’re walking, and because you (researchers) are monitoring it there’s a tendency to have the guilty conscience if you’re not doing it. Therefore, you tend to meet that (step) goal*.

(Male, 70, prostate cancer)

Another participant commented on the importance of personal connection with the researchers:

*Well I think you’re all; you just gave us that extra enthusiasm to stick with it ... I personally feel I got to know you well… and I think that’s generally a feeling that you were all a great help to us. So that’s … having somebody there that you can relate to I think*.

(Female, 78, breast cancer)

Other participants described feelings of responsibility for their health due to the encouragement and support received:

*I think you’ve brought it to the forefront about your health and that there are actually people that are trying to help people look after themselves, and so you feel as though well, they’re putting in their time, you’ve got to do your time. There’s no good just dragging yourself and saying oh yes, I can’t be bothered, you know. So, if everybody else is willing to do their part, I think you’ve got to do yours*.

(Female, 78, breast cancer)

All participants liked their step goals to be set by researchers, and this was a highly motivating aspect for them. When participants were asked if they would have liked more control over their step goals, the answer was unanimously ‘no’. All participants enjoyed receiving their step goals until the end of the program. One participant highlighted the need for external regulation by commenting that he did not think he would have been as motivated if responsibility of step goals was shifted to him:

*I probably wouldn’t be disciplined enough to faithfully follow it through, I think I need that outside input, in my case*.

(Male, 65, prostate cancer)

Similarly, another participant commented that if participants were to set their own goals it would not have been as motivating to achieve higher step counts:

*No that would make people too lazy. I think when someone sets you something to do no matter how many steps, you do it, otherwise you just say oh right and you do five today when you’ve been setting a person 12 so I think having the STRIDE people set the goal is very good*.

(Male, 70, prostate cancer)

*It’s a challenge, if I did it myself I might set too low a goal, whereas you’re creating it from facts, from figures and if I can’t achieve it that’s okay but I think it’s based better, based more scientifically if you do it rather than me just thinking oh well only feel like doing so many steps today so that’s all I’ll do*.

(Female, 60, breast cancer)

However, this participant then went on to say that since the program finished, she no longer needed to count steps because she knows the amount of walking needed. This illustrates her competence of physical activity maintenance post-intervention.

*I don’t need to count the steps, because I know the, the amount of time that I’m walking. I think before the step program I was probably walking about 40 min, whereas now our minimum walk is an hour up to even close to two hours*.

(Female, 60, breast cancer)

Many participants appreciated receiving support from their family and friends. Several participants enjoyed their partners joining them on their walks, and this seemed to be an important incentive:

*Joan (pseudonym) and I doing it together was great because when we started, we were off, and we’d go for an hours walk or whatever and, and it was good to get away and talk and, and do that part of it, I thought it was great*.

(Male, 67, face cancer)

In a similar vein:

*… it was good in the fact that we were both talking to one another about what we’re eating and how we’re walking and being part of the program together was good*.

(Female, 62, breast cancer)

##### (3) Website Factors

A frequent theme throughout the interviews was that the step log and graph on the STRIDE website were powerful self-monitoring and self-motivating tools. It helped some participants keep a routine. Entering their step counts and seeing their weekly averages presented visually on a graph were useful features to monitor their progress throughout the program:

*I enjoy going on and typing in my steps and seeing the graph—that’s a physical motivator for me ... And I’ve done it on a graph on my fridge and stuck it on things like that … that does motivate me … that does actually help me stay on track*.

(Female, 62, breast cancer)

The fact that the step goals were tailored to each participant was also appreciated. One participant said that he would recommend the program to others because the program ‘*gives you goals, and it gives you a target to work for; it’s fairly personalized*’ (Female, 62, breast cancer).

#### 3.2.2. Perceived Barriers

Factors that hampered participants’ ability to stay active were categorized under four main themes: Physical, psychosocial, environmental, and organizational barriers.

##### (1) Physical Limitations

Physical factors including injury, fatigue and mobility limitations that negatively affected the number of steps participants were able to achieve. A few participants mentioned that they had to curtail their walking due to ‘flare-ups’ or body soreness. Some of the injuries mentioned were previous injuries that were exacerbated from increased walking or were unrelated to the walking. One participant mentioned her physical limitations:

*My joints are just so sore and so all I want to do is sit down and put my feet up. …I really like to try and get out before lunchtime if I can, any later than that, I’ve done it, but it’s a real struggle and quite uncomfortable*.

(Female, 60, breast cancer)

Similarly, another female participant commented:

*I had my legs packed-up, that sort of hinders me a bit*.

(Female, 78, breast cancer)

##### (2) Psychosocial Factors

Participants spoke of attitudinal and emotional factors that interfered with their physical activity routines:

*… some days you had a bit of a downer where you didn’t feel like … you didn’t feel good enough*.

(Female, 78, breast cancer)

Some psychosocial barriers mentioned were unique to rural areas, particularly beyond townships. Some participants mentioned that it is unenjoyable walking in rural areas due to lack of excitement and social opportunities:

*It was not very exciting at my place—up the road, and back down the road. It’s not like in town where you can—you walk around, you see people, you can make a comment. Out there it’s, other than the occasional kangaroo that passes you and waves, there’s nothing else that will excite you*.

(Male, 70, prostate cancer)

He also mentioned the social isolation of living out of towns as a barrier to walking:

*No one comes and visits you because you’re 20 k’s out of town and you’re also six kilometers off the bitumen road. The isolation is a barrier*.

(Male, 70, prostate cancer)

Several participants discussed the difficulties in maintaining their motivation to walk after the program had finished. One participant mentioned that she had reduced her physical activity levels post program because she felt as though she lacked external encouragement and organization:

*Perhaps I’m better off with somebody organizing me and telling me what to do … (I) get a bit slack if I’m just left to do it myself*.

(Female, 78, breast cancer)

Another participant commented that after the program had finished, there was a lack of follow-up from researchers which made it difficult for her to stay motivated to maintain her walking:

*I guess since the project—since the three months has finished there has been really nothing from you guys to us, so I’ve been in control of it and that hasn’t really worked like it did when the project was happening.*

She suggested that tools or mechanisms to increase independence would be beneficial.

*So, I guess some direction in how to manage it. Perhaps some tools to be able to wean off being looked after for that three months and how to now shift into looking after yourself maybe*.

(Female, 60, breast cancer)

##### (3) Environmental Barriers

Participants frequently cited extreme weather as a major impediment to their daily walking:

*In the middle of summer it’s bloody hot and there’s no shade*.

(Male, 67, face cancer)

*Really bad weather … really strong winds is a barrier; I mean that danger would be a barrier*.

(Female, 60 years, breast cancer)

*It was a winter thing and you’d look out and it was just pouring, you couldn’t go out in that weather. So those days you were only doing 4 or 500 steps you know, which just wasn’t enough because it was too cold to go anywhere*.

(Female, 78, breast cancer)

Poor walking environments and safety issues (dirt roads, bush scrub, farms, bush flies, and snakes) in rural areas were also identified as barriers as they were not conducive to walking:

*… the only place we can walk is, is along an unmade, a dirt road and with crops on both sides … and there’s always potential for snakes*.

(Male, 67, face cancer)

One participant said he was reluctant to walk because of bush flies (commonly found in rural areas of Australia):

*Well actually the only thing I really disliked was the flies on the walks, the bush flies. You don’t get them like we do out in the bush. So that was the most annoying thing of the whole exercise … it’s a rural barrier to it. It kind of puts you off unless you’re wearing a fly veil*.

(Male, 70, prostate cancer)

This participant also mentioned the environment and physical isolation of living out of town as being barriers to walking:

*I can only walk … down a road … And the only thing that keeps you on a program is having the goals and the commitment to doing them and I guess somewhere to walk and if it’s more exciting than down a (road) paddocks on either side, which have been ploughed. So, my setting, my environment I don’t think was conducive to actually encouraging walking*.

(Male, 70, prostate cancer)

Despite the above perceptions, some participants demonstrated high barrier self-efficacy when they expressed capabilities to walk in the face of barriers:

*We’ve got a veranda around our house, and when I couldn’t get out because it was pouring with rain, I would just walk walk, and walk, and walk, around, and around, and around the veranda*.

(Female, 62, breast cancer)

*I really don’t mind if I get a bit wet, if it’s, if it’s hot, get up a bit earlier go or do the walk that’s got more shade, there’s some areas that are shadier than others, take water*.

(Female, 60, breast cancer)

##### Organizational Barriers

Several participants reported timing constraints which made it difficult to schedule their walking. The following quotes represent the overarching themes of lack of time and competing priorities:

*I’d walk, I’d made myself an hour in the morning and an hour at night, either before or after work … and I thought, well I can’t just keep walking and not get anything else done in my life, there’s more to it*.

(Female, 62, breast cancer)

*There were a couple of days, where we drove to Adelaide and back, well there’s six hours out of the day to start with, because we went down for medical appointments. So, there’s just no time for walking, if you’re going to spend six hours in a car*.

(Male, 65, prostate cancer)

*Probably priorities, if I had things I needed to do, they took priority, rather than trying to think about—am I doing my steps*.

(Male, 65, prostate cancer)

Another participant mentioned her challenge to balance work and physical activity:

*I prefer to walk in the morning, my joints aren’t very good later in the day, so if I was busy all day—for instance during the school holidays when I’m working all day at Vacation Care, if I don’t get home till 5:30 pm, 6:00 pm I’m too tired to go for a walk*.

(Female, 60, breast cancer)

## 4. Discussion

Maintaining physical activity after a cancer diagnosis is an important health priority given its numerous associated physical and psychosocial benefits [29]. However, cancer survivors living in rural and remote areas are more sedentary and have poorer health outcomes compared to their urban counterparts. Limited physical activity engagement and poorer outcomes may be primarily attributed to geographical isolation; the burden of travel distances, and limited access to services and facilities [12]. Certain facilities, such as swimming pools and gymnasiums, are less readily available in rural areas compared with metropolitan areas [8]. Other contributing factors common to the rural environment include social isolation, lower socioeconomic status, concerns about privacy in small community settings, financial issues, demands of farming, and possible reluctance to accept services due to a stoic approach to life [28,29,30]. Research that unveils factors that aid or inhibit cancer survivors in rural areas to maintain physical activity is important for the development and implementation of effective interventions among this vulnerable population.

The purpose of this study was to explore the motivators and barriers to maintaining physical activity changes after a walking intervention among rural cancer survivors. There were several factors that inhibited rural cancer survivors’ physical activity participation and maintenance, including physical factors (e.g., fatigue, physical health, and mobility limitations), features of the environment (e.g., poor weather, safety, and geographical isolation) and organizational barriers (e.g., time constraints and competing priorities). These findings are comparable with findings of other physical activity interventions that have identified exercise barriers among cancer survivors [28,29,30,31]. For example, a study on exercise barriers in colorectal cancer survivors concluded that the most common barriers to exercise were fatigue and lack of time [30]. Among a population of breast and prostate cancer survivors, the most commonly reported exercise barriers were ‘too busy’, ‘lack of willpower’, and ‘don’t like to exercise in bad weather’ [31], findings that are consistent with the current study. Contrary to other studies (i.e., a study among breast cancer survivors [32] that reported unique cancer-related barriers to physical activity), this study did not find any cancer-specific barriers. This may be because the cancer survivors in the current study had varying cancer sites (prostate, breast, and face) and the time since diagnosis ranged from 4–19 years (mean = eight years) as opposed to the majority of participants in other studies being five years since diagnosis.

The findings of this study also show that cancer survivors are motivated to engage in physical activity for three primary reasons: Physical and psychological benefits (weight loss, feeling better, increased fitness, and improved body image), social support (obligation, encouragement from research team, appreciating support from the research team, enjoying connecting with others), and website factors (tailored step goals, graphic visualization of progress on website). Again, these findings are congruent with other similar studies. For example, a qualitative study with breast cancer survivors found that the primary reasons for exercise were experiencing improved health, preventing illness, enjoyment and fun, social contact and support, body image management, and moral obligation [32].

According to Self-determination Theory [25], maintenance of behaviors over time requires that participants internalize skills and values for change and develop self-determination. The theory also posits that by maximizing an individual’s experience of autonomy, competence, and relatedness, the regulation of health-related behaviors is more likely to be internalized, and behavioral change will be better maintained [33,34]. Overall, it seems that STRIDE was unsuccessful in creating autonomous motivation. It appears that the majority of the reported motivation was from extrinsic sources in that participants were highly motivated by research personnel and were dependent on daily step goals being set for them until the end of the program. This suggests a lack of autonomy; when the external sources of motivation ceased upon program completion, participants lacked the skills and ability to maintain their regular walking routines. Some participants described feelings of competence when they were able to achieve step goals and no longer needed to count steps on the pedometer because they knew how far they should be walking. However, several participants also expressed lack of competence through their inability to set goals themselves after the intervention had completed because they ‘might set too low a goal’, would be ‘better off with someone organizing me and telling me what to do’, and that they might ‘get a bit slack’ if left to do it themselves.

Research has suggested that individuals are more likely to maintain physical activity post interventions when they report intrinsic goals [35,36]. In line with Self-determination Theory, Ingledew and Markland [37] clarified that physical activity interventions should move away from a focus on weight loss (an externally regulated motive) and instead encourage identified regulation such as health and fitness types of motive. The challenge for researchers is to identify the most effective methods to increase autonomous motivation for exercise among different populations.

Based on the findings of the current study, future studies should embrace a comprehensive ecological approach, including Self-determination Theory, which focuses on multiple factors including interpersonal, organizational and environmental influences to promote physical activity participation and long-term adherence. From an organizational level, researchers could incorporate strategies into the intervention to help participants become more self-sufficient in their physical activity behavior change. Researchers could implement and investigate the effects of follow-up strategies (e.g., regular telephone calls, regular supportive text messages, or monthly catch-ups) to help make participants more autonomous and independent of external agencies. Participants could maintain the use of an accessible daily log that can generate step goals and provide graphical feedback. Healthcare providers may be suitably placed to provide a basic level of support (compared to what was received in the intervention) to review pedometer-based self-monitoring records and to encourage participants to seek support in maintaining their walking routines. Beyond the clinician-participant relationship, rural local governments could create opportunities for walking through town planning efforts.

This study has several limitations. The sample was relatively homogenous. In particular, all of the participants were Caucasian, and 50% were breast cancer survivors and 40% were prostate cancer survivors. Thus, the generalizability of results to other subgroups of cancer survivors is limited. Participants were from only two rural towns in South Australia. However, they were purposively selected based on their geographical difference (inland vs. coastal) in order to obtain a broader and more in depth understanding of the potential barriers and motivators associated with different physical environments. Participants were only interviewed on one occasion after the STRIDE intervention (three months after program follow-up). Therefore, it was not possible to conclude how the participants’ perceptions of physical activity maintenance may change over longer periods of time. Despite these limitations, this study makes an important contribution to the current field by revealing the physical activity perceptions of participation and maintenance of a vulnerable and understudied population.

## 5. Conclusions

This study identified several perceived motivators and barriers to engagement and maintenance of regular walking after a 12-week, pedometer-based walking program among rural cancer survivors. Rural cancer survivors in this study found physical and psychological benefits, social support and connections, and features of the website as motivators to engage in physical activity beyond the intervention. Participants found physical limitations, psychosocial, environmental, and organizational barriers as impediments to engaging in physical activity beyond the intervention. Researchers and practitioners aiming to promote sustained physical activity among rural cancer survivors should focus on encouraging autonomy and relatedness in line with the Self-determination Theory. Such strategies can help participants progress from extrinsic to more intrinsic motivation for sustained behavior change post-intervention. Future research should explore the effectiveness of STRIDE and similar interventions in larger rural samples and with longer-term follow-up to more fully identify predictors of maintenance of behavior change. In addition, more research on physical activity adherence is needed to determine the optimal timing of follow-up strategies and which approaches are most effective in promoting sustained physical activity participation.

## Figures and Tables

**Table 1:** Summary of participant characteristics.

Characteristics	
Age (y)	
Mean	74.0
SD	5.5
Range	60–78
Sex (*n*)	
Male	5
Female	5
Ethnicity (*n*)	
Caucasian	10
Cancer type (*n*)	
Breast	5
Prostate	4
Face	1

Note: *n* = number; y = year.

**Table 2:** Motivators and barriers to walking.

Theme		Sub-Theme
Motivators	Physical/psychological benefits	Increased fitness
		Weight loss
		Feeling better
		Improved body image
	Social support	Obligation to research team
		Appreciated support and encouragement from research team
		Enjoyed connecting with others
	Website factors	Tailored step goals
		Graphic visualization of progress on website
Barriers	Physical limitations	Fatigue
		Pain
		Chronic illness
	Psychosocial barriers	Social isolation
		Lack of motivation/encouragement
	Environmental barriers	Poor weather conditions
		Poor walking environment
		Safety issues
		Geographical isolation
	Organizational barriers	Time constraints
		Competing priorities
